# Human Serum Albumin Aggregation/Fibrillation and its Abilities to Drugs Binding

**DOI:** 10.3390/molecules25030618

**Published:** 2020-01-31

**Authors:** Małgorzata Maciążek-Jurczyk, Kamil Janas, Jadwiga Pożycka, Agnieszka Szkudlarek, Wojciech Rogóż, Aleksandra Owczarzy, Karolina Kulig

**Affiliations:** 1 Department of Physical Pharmacy, Faculty of Pharmaceutical Sciences in Sosnowiec, Medical University of Silesia, 40-055 Katowice, Poland; kamil.wojciech.janas@gmail.com (K.J.); jpozycka@sum.edu.pl (J.P.); aszkudlarek@sum.edu.pl (A.S.); wrogoz@sum.edu.pl (W.R.); karolina.k94@gmail.com (K.K.); 2 Independent Public Clinical Hospital No. 1 in Zabrze, Medical University of Silesia, 40-055 Katowice, Poland; olaowczarzy@gmail.com

**Keywords:** human serum albumin, aggregation/fibrillation, spectroscopic methods

## Abstract

Human serum albumin (HSA) is a protein that transports neutral and acid ligands in the organism. Depending on the environment’s pH conditions, HSA can take one of the five isomeric forms that change its conformation. HSA can form aggregates resembling those in vitro formed from amyloid at physiological pH (neutral and acidic). Not surprisingly, the main goal of the research was aggregation/fibrillation of HSA, the study of the physicochemical properties of formed amyloid fibrils using thioflavin T (ThT) and the analysis of ligand binding to aggregated/fibrillated albumin in the presence of dansyl-l-glutamine (dGlu), dansyl-l-proline (dPro), phenylbutazone (Phb) and ketoprofen (Ket). Solutions of human serum albumin, both non-modified and modified, were examined with the use of fluorescence, absorption and circular dichroism (CD) spectroscopy. The experiments conducted allowed observation of changes in the structure of incubated HSA (HSA_INC_) in relation to nonmodified HSA (HSA_FR_). The formed aggregates/fibrillation differed in structure from HSA monomers and dimers. Based on CD spectroscopy, previously absent β-structural constructs have been registered. Whereas, using fluorescence spectroscopy, the association constants differing for fresh and incubated HSA solutions in the presence of dansyl-amino acids and markers for binding sites were calculated and allowed observation of the conformational changes in HSA molecule.

## 1. Introduction

Human serum albumin (HSA) is the most common protein in the human body. HSA is present in plasma (60% of protein mass), lymph, saliva, cerebrospinal and interstitial fluid [1,2]. It is easily soluble in salt solutions in the pH range of 4.0 to 8.5, and in an aqueous environment [3]. The secondary structure of HSA is constituted by 67% α-helix, 23% stretched chain, 10% β-sheets and bends. However, β-pleated sheet structures are not present. The structure of albumin has been divided into three domains (I, II and III) [4], and each of domains has been divided into two subdomains, A and B, defining the ability to bind individual substrates by respective domains. Using fluorescent probes Sudlow et al. classified the high-affinity binding sites for drugs into sites I and II, called warfarin (also for phenylbutazone) and benzodiazepine (also for ketoprofen) binding sites, respectively [5]. They are located in the hydrophobic cavities of the molecule, subdomain IIA and IIIA, respectively [6]. A few years later Yamasaki et al. proposed novel nomenclatures for binding site I: regions Ia, Ib and Ic [7]. Binding region of phenylbutazone corresponds to the region Ia and Ib. The most important function of proteins is to maintain oncotic pressure and buffering function as well as binding capacity. Depending on pH (1.2–9.0), HSA undergoes transformation and occurs in different isoforms: E, F, N, B, A [8]. Outside the limits of pH values, the secondary and tertiary structure of HSA changes, causing its unfolding and an increase in the β-plated sheets, replacing α-helical structure [9,10].

In spite of α-helical structure, many proteins, i.e., HSA, present an abnormal structure and metabolism associated with a strong tendency to self-aggregation into a polymeric amyloid fibril structure, and this ability is a generic feature of the polypeptide chains [11,12]. The aggregation process is composed of two parts. The first step is reversible nucleation and proteins are sequentially added to a growing nucleus in a thermodynamically reversible manner. The second part of the process occurs after the growing nucleus reaches a critical mass. Then there are further irreversible additions of protein molecules to the nucleus. This leads to the formation of a large aggregate that is very stable, and there is no evidence for an equilibrium between aggregated state and correctly folded state [13]. HSA can form aggregates resembling those in vitro formed from amyloid at physiological pH (neutral and acidic). Amyloid fibrils are peptide or protein aggregates that form under certain conditions in vitro or in vivo. Amyloid fibrils are formed by soluble proteins that are assembled to form insoluble resistant-to-degradation fibers. They refer to abnormal fibrous regions found in organs and tissues, including different parts of the eye. Cataracts in the lens and retinitis pigmentosa in the retina are the best studied ocular conformational diseases [14]. These composites are structurally dominated by β-sheet structure. Amyloid fibril plaques can be found in brain tissue of Alzheimer patients so they are associated with neurodegenerative diseases belonging to amyloidosis [15]. The formation of the fibrils can be induced by increased temperature, ionic strength, protein concentration or chemical denaturants [16,17,18], and the fibrils can further associate in other more complex structures such as fibrillar gels, plaques, or spherulitic structures [17]. The phenomenon of protein aggregation/fibrillation results from partial unfolding of the tertiary structure and conformational changes of the secondary structure [19]. It is important to understand the mechanism of aggregation/fibrillation prevention and to design suitable inhibitors. The process of HSA fibrillation has been well documented [20,21,22,23,24,25,26,27,28]. Fibrillar aggregates (amyloid fibrils, protein fibrils) are insoluble and heterogenous, characterized by a cross-stretched β-structure and the ability to bind dyes such as thioflavin T (ThT) and Congo red. The Congo red spectrophotometric method could be used as a primary test for the evaluation of protein fibrils [29], while the benzothiazole dye, thioflavin T (ThT) assay, is widely used for the in vitro quantitative assessment of amyloid fibrils [30,31]. 

Phenylbutazone (Phb) and ketoprofen (Ket) belong to the group of nonsteroidal anti-inflammatory drugs (NSAIDs). Phb is a recognized drug (marker) that specifically binds to Sudlow’s site I (IIA subdomain), while Ket binds specifically to Sudlow’s site II (IIIA subdomain). Phenylbutazone is a derivative of pyrazolone, whereas ketoprofen belongs to phenylpropionic acid derivatives. 

Human serum albumin (HSA) in the human body occurs in the form of individual molecules or dimers [32]. Under favorable conditions, like the monomer, HSA dimers forms aggregates and fibrillates, changing its structure through changes in the ligand binding sites and precautions are required during a therapy. The aim of this study was to evaluate changes in Sudlow I and II sites using dansyl amino acids and the ability of HSA to bind non-steroidal anti-inflammatory drugs (NSAIDs) to these sites depending on the state in which the protein molecule is present. Due to the fact that commonly used methods of amyloid fibril structure determination are difficult (X-ray fiber diffraction, electron microscopy, solid state nuclear magnetic resonance, electroparamagnetic resonance [33,34,35,36,37]), fluorescence, absorption and circular dichroism (CD) spectroscopy have been applied; the study of binding sites markers can be used as a model for aggregation processes of serum albumins.

## 2. Materials and Methods 

### 2.1. Chemicals

Human serum albumin (HSA), ketoprofen (Ket), dansyl-l-glutamine (dGlu) were purchased from MP Biomedicals, Inc. Illkirch, France, Phenylbutazone (Phb) was obtained from Sigma Chemical CO., St Louis, MO, USA, dansyl-l-proline (dPro) from Fluka Chemie AG, Buchs, Switzerland, thioflavin T (ThT) ultra-pure grade from AAT Bioquest, Sunnyvale, CA, USA, ethanol absolute Uvasol^®^ for spectroscopy from Merck KGaA, Darmstadt, Germany. 

### 2.2. Methods

#### 2.2.1. Sample Preparation

HSA solution at 3 × 10^−4^ mol·L^−1^ was incubated at pH 2.0 in HCl for 2 h at 24–25 °C (protein isoform E), then filtered using a sterile Millex-GP syringe filter with 0.22 µm pores. After 2 h of incubation at room temperature the HSA solution was divided into two parts. The first part of HSA solution was used to prepare spectroscopic studies (HSA_FR_), and the second part was incubated for 7 or 9 days at 37 °C (HSA_INC_). After incubation no precipitates were observed and all solutions were clear. All solutions have been diluted to 5 × 10^−6^ mol·L^−1^ in 0.01 mol·L^−1^ phosphate buffer at pH 7.4.

Thioflavin T (ThT)–HSA system: A stock solution of thioflavin T (ThT) at 1 × 10^−3^ mol·L^−1^ concentration was prepared in 0.01 mol·L^−1^ phosphate buffer at pH 7.4 and diluted to 1 × 10^−5^ mol·L^−1^. HSA concentration was 1 × 10^−6^ mol·L^−1^, λ_ex_ 440 nm.

Dansyl-l-glutamine (dGlu) and dansyl-l-proline (dPro)–HSA systems: Stock solutions of dansyl-l-glutamine (dGlu) and dansyl-l-proline (dPro) at 5 × 10^−3^ mol·L^−1^ concentration were prepared in methanol. HSA_FR_ and HSA_INC_ solutions at 3 × 10^−4^ mol·L^−1^ were diluted to 1 × 10^−5^ mol·L^−1^ by 0.01 mol·L^−1^ phosphate buffer (pH 7.4). HSA_FR_ and HSA_INC_ samples were titrated by dGlu (5 × 10^−3^ mol·L^−1^), λ_ex_ 350 nm, emission fluorescence spectrum range 450–550 nm. For each sample an absorbance at 350 nm was registered. The measurements have been repeated for dPro–HSA_FR_ and dPro–HSA_INC_ systems.

Phenylbutazone (Phb) and ketoprofen (Ket)–HSA systems: Stock solutions of phenylbutazone (Phb) and ketoprofen (Ket) at 2.5 × 10^−3^ mol·L^−1^ concentration were prepared in methanol. HSA_FR_ and HSA_INC_ solutions at 3 × 10^−4^ mol·L^−1^ has been diluted to 5 × 10^−6^ mol·L^−1^ by 0.01 mol·L^−1^ phosphate buffer (pH 7.4) and titrated by Phb (2.5 × 10^−3^ mol·L^−1^), λ_ex_ 275 nm and λ_ex_ 295 nm. The measurements have been repeated for Ket–HSA_FR_ and Ket–HSA_INC_ systems.

#### 2.2.2. Emission, Synchronous and Absorption Spectra Measurements

Emission and synchronous fluorescence spectra were taken using spectrofluorimeter JASCO FP-6500, Hachioji, Tokyo, Japan with quartz cuvette at 10 mm pathlength, T = 20 °C, error apparatus ±1.5 nm.

Parameters for emission spectra of dGlu–HSA and dPro–HSA systems: range 450–550 nm, λ_ex_ 350 nm. Parameters for emission spectra of Phb–HSA, Ket–HSA systems: range 285–400 nm at λ_ex_ 275 nm and 305–400 nm at λ_ex_ 295 nm. Parameters for synchronous emission spectra registered for fresh (HSA_FR_) and incubated (HSA_INC_): for ∆15 nm range of emission 280–330 nm, λ_ex_ 265–315 nm (Tyr excitation), and for ∆60 nm range of emission 310–380 nm, λ_ex_ 250–320 nm (Trp excitation).

The emission spectrum (scattering spectrum) of the solvent used to obtain the HSA solution (in 0.01 mol·L^−1^ phosphate buffer, pH 7.4) was subtracted from each of the obtained spectra.

Due to the absorption of light at both excitation and emission wavelengths (inner filter effect, IFE), a correction of fluorescence intensity is required. The absorbance measurements at the wavelength used to excite fluorophores fluorescence were made using a JASCO V-530 spectrophotometer and for the inner filter correction Equation (1) has been used [38]:

(1) $$F_{\mathrm{cor}}\;=\;F_{\mathrm{obs}}\cdot 10^{\frac{A_{\mathrm{ex}}+A_{\mathrm{em}}}{2}}$$

where: F_cor_ and F_obs_ are corrected and observed fluorescence (after subtraction the scattering spectrum of solvent), respectively, A_ex_ and A_em_ are the absorbance at the excitation and emission wavelength, respectively.

The association constant K_a_ in ligand–protein system has been determined from the Scatchard equation [39]:

(2) $$\frac{r}{L_{f}}=nK_{a}-K_{a}\cdot r$$

where: n – is a number of binding sites classes, r is a number of ligand moles bound to 1 mole of protein; $r=\frac{L_{b}}{P_{t}}$ where L_b_ is bound ligand concentration and P_t_ is total protein concentration, L_f_ is a free ligand concentration.

When the Scatchard curve is not straight, this indicates the presence of more than one class of binding site. For two classes of binding sites in HSA structure, the binding isotherms were drawn employing non-linear regression analysis according to Equation (3) and the association constants K_a1_ and K_a2_ and the number of binding sites n_1_, n_2_ were calculated:

(3) $$r=\frac{n_{1}\cdot K_{a1}\left[L_{f}\right]}{1+K_{a1}\left[L_{f}\right]}+\frac{n_{2}\cdot K_{a2}\left[L_{f}\right]}{1+K_{a2}\left[L_{f}\right]}$$

#### 2.2.3. Circular Dichroism (CD) Measurements

Circular dichroism (CD) is an absorption spectroscopy method based on the differential absorption of left and right circularly polarized light [40]. The CD spectrum of far ultraviolet (far-UV CD) proteins below 250 nm reflects the secondary structure of the protein, i.e., the α-helical structure, β-sheet, β-turn and unstructured elements. In the CD spectrum, all α-proteins show a strong double minimum at 222 nm and 208–210 nm and a stronger maximum at 191–193 nm. All β-proteins usually have a single negative band in the 210–225 nm wavelength range and a stronger single positive band in the 190–200 nm wavelength range, whose intensities are much lower than those for α-proteins.

The mean residue ellipticity [Θ]_MRW_ is represented as:

(4) $$[\Theta {]}_{\mathrm{MRW}}\;=\frac{\mathrm{MRW}\cdot \Theta }{10\cdot l\cdot m}\;\left[\deg\cdot {\mathrm{cm}}^{2}\cdot {\mathrm{dmol}}^{-1}\right]$$

where: Θ is observed ellipticity for a given wavelength [deg], m is the concentration in g/cm^3^, and l is the pathlength in cm, MRW is a mean residue weight (MRW_HSA_ = 113.7 Da) [41,42].

Circular dichroism (CD) spectra of 3 × 10^−6^ mol·L^−1^ HSA_FR_ and HSA_INC_ were recorded using JASCO J-1500 spectropolarimeter (Hachioji, Tokyo, Japan). The measurements were made at 20 °C, in quartz cuvettes with an optical path equal to 2 mm. The spectra were recorded in the wavelength range from 200 to 250 nm (secondary structure image). The accuracy of the wavelength measurement was ±0.1 nm and the wavelength repeatability was ±0.05 nm.

### 2.3. Statistics

The results of the study were expressed as a mean ± relative standard deviation (SD) from three independent experiments. Linear regression was analyzed using OriginPro version 8.5 SR1 software (Northampton, MA, USA) by fitting experimental data to the corresponding equation.

## 3. Results and Discussion

### 3.1. In-Vitro Quantitative Assesment of Amyloid Fibrils

Amyloid fibrils are peptide or protein aggregates formed under certain conditions in vitro or in vivo. Soluble proteins that are assembled to form insoluble, resistant-to-degradation fibers, can form amyloid fibrils. They refer to abnormal fibrous structures found in organs and tissues. These composites are structurally dominated by β-sheet structure. Amyloid fibril plaques can be found in brain tissue of Alzheimer patients, so they are associated with neurodegenerative diseases belonging to amyloidosis. In addition to Alzheimer’s disease, amyloidosis includes the spongiform encephalopathies and type II diabetes, progressive disorders with associated high morbidity and mortality.

Benzothiazole dye–thioflavin T (ThT) was used to quantify amyloid fibrils in vitro. The interaction of ThT with globular proteins, and even with their amorphous aggregates is insignificant when compared to the interaction with protein fibrils [43]. ThT becomes incorporated with the amyloid fibrils, has a maximum at 450 nm and is dependent on solvent polarity [44]. However, the position of ThT fluorescence spectra depends on the polarity of solvent to a much lesser extent than its absorption spectrum. Maskevich et al. showed that when excited at 440 nm, ThT has emission with maxima at 493 and 478 nm in water and fibrils, respectively.

**Figure 1:**
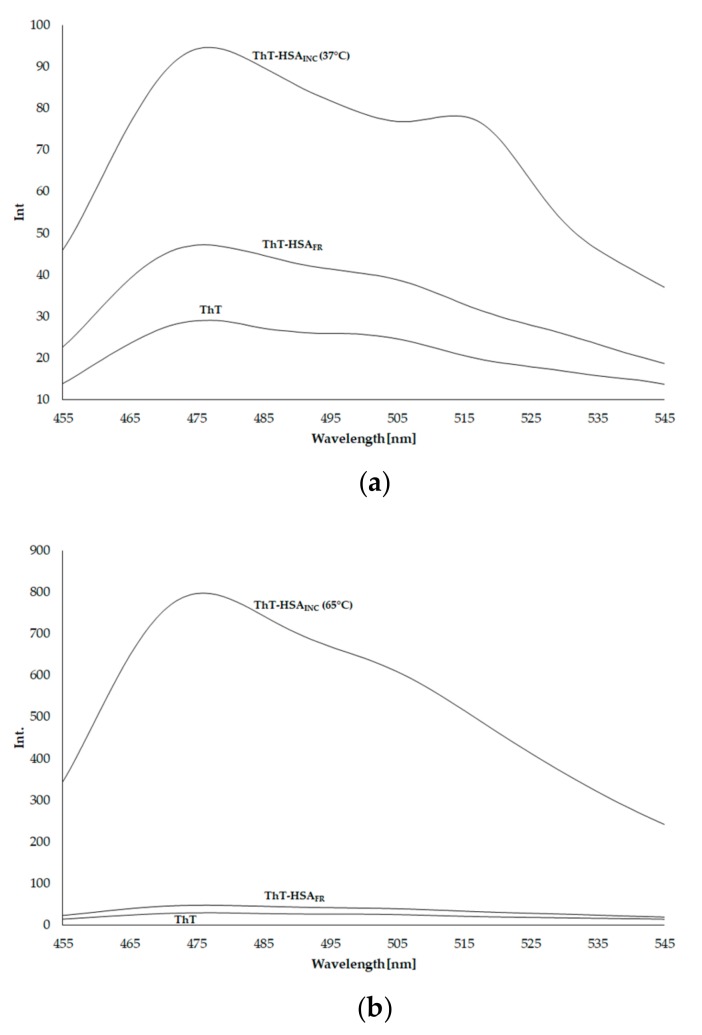
Emission fluorescence spectra of thioflavin T (ThT) (1 × 10^−5^ mol·L^−1^) in the presence of unmodified human serum albumin (HSA_FR_) and incubated human serum albumin (HSA_INC_) at (**a**) 37 °C for 9 days and (**b**) 65 °C for 7 days; HSA concentration 1 × 10^−6^ mol·L^−1^, λ_ex_ 440 nm.

Figure 1 shows thioflavin ThT fluorescence spectra (ThT), in the presence of fresh (ThT–HSA_FR_) and incubated (ThT–HSA_INC_) at both 37 °C and 65 °C. Data are collected in Table 1.

α-helical HSA with destabilized conformation at 65 °C forms fibrils. HSA fibrils are also formed at physiological temperature, i.e., 37 °C, although their quantity is much smaller, assuming that the ThT fluorescence intensity is proportional to the concentration of protein fibrils. Most previously published work on the aggregation process used heat to induce fibrils (at 57–65 °C); however the aggregation/fibrillation process under physiological conditions is more relevant to the fibrils formed in natural cellular environments. Juarez and Taboada reported that longer incubation times lead to more complex morphological variability of amyloid mature fibrils (i.e., long straight fibrils, flat-ribbon structures, laterally connected fibers, etc.) [45].

Thioflavin T (ThT) in a solution of 0.01 mol·L^−1^ phosphate buffer at pH 7.4 excited at 440 nm wavelength fluoresces slightly. The fluorescence intensity at 477 nm is 29.032. In the presence of amyloid fibrils of HSA_FR_, ThT fluorescence increases to 47.185 (an increase in fluorescence intensity about 1.625 times). After incubation of HSA at 3 × 10^−4^ mol·L^−1^ concentration (pH 2.0), for 9 days at 37 °C, ThT (1 × 10^−5^ mol·L^−1^) fluorescence in the presence of diluted HSA to 1 × 10^−6^ mol·L^−1^ increases to 94.624 (2-fold increase in fluorescence). With the increase of incubation temperature of HSA_INC_ from 37 °C to 65 °C (7 days incubation, similar to the Taboada et al. method [11]), an increase in the ThT–HSA fluorescence to 797.720 (22.51 times higher) has been recorded. By the increase of incubation temperature from 37 °C to 65 °C the intensity of ThT fluorescence increases 9 times. Pandey et al. studied the effect of temperature and solvent on fibrillation of HSA [18]. They reported that based on the ThT binding both ethanol concentration and temperature play crucial roles in the transformation of native HSA into its fibrillar analogue at pH 7.0. ThT intensity gradually becomes more significant at higher temperatures.

The increase in dye signal suggests the existence aggregate species [46]. It is noteworthy that the ability of proteins to form ordered fibrillar cross-β structures is inextricably linked to the nature of the protein backbone [47,48] and is independent of native state structure [46]. Holm et al. reported, that it should be possible to find conditions that are destabilizing for the native state to allow the protein to explore alternative conformations and lock on to the stable β-sheet aggregated state [48]. As Vetri et al. concluded, aggregation processes in serum albumin (human, bovine) follow different aggregation pathways strictly affected by hydrophobic interactions modulated by pH [49]. Changes in protein secondary structure turn out to be the driving mechanism of the observed aggregation and they progress in parallel with the growth of ThT emission intensity. This suggests a mutual stabilization of elongated protofibril-like structures and of protein conformational and structural changes, which lead to a more rigid and ordered structures [50]. Although the mechanism of ThT interaction with amyloid fibrils is still poorly understood, it is widely accepted that ThT molecules intercalate inside the furrows (channels) between exposed to solvent side chains of amyloid fibrils located parallel to the long axis of fibrils. However, the interaction between ThT and amyloid fibrils is stoichiometric, saturated, and the fluorescence of the ThT–amyloid fibril system provides an accurate quantitative assessment of amyloid fibril formation [43,51]. Taboada et al. used Congo red as a dye that is able to bind with fibrils. They observed a characteristic red-shift of Congo red optical absorption from 490 to 540 nm together with the characteristic green birefringence. X-ray diffraction was also used and two reflections: a larger one at 4.8 Å corresponding to the inter-strand spacing in the β-sheet and a second one with lower intensity at 10 Å, characteristic of multilayer β-sheet were observed [11]. Moreover these changes also involve the presence of different structural intermediates in the aggregation pathway, such as oligomeric clusters (globules), bead-like structures, and ring shaped aggregates [45]. Formation of amyloid fibrils not only from human serum albumin but also β-lactoglobulin were a point of interest for many scientists. Jordens et al. studied structural amyloid-like β-lactoglobulin fibrils incubated in ethanol–water mixtures after their formation in water [52]. Based on the AFM imaging they observed that with longer incubation time the contour length of wormlike structures increases, whereas the amount of original fibrils decreases in mixtures with high ethanol concentrations.

### 3.2. Absorption Spectra of Human Serum Albumin (HSA)

**Figure 2:**
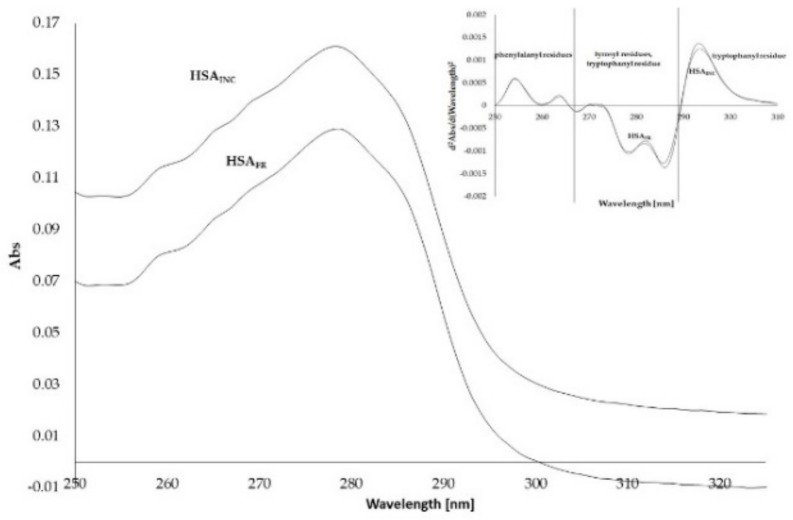
Absorption spectra of unmodified human serum albumin (HSA_FR_) and incubated human serum albumin (HSA_INC_) at 5 × 10^−6^ mol·L^−1^ concentration. In the insert: second derivative of 5 × 10^−6^ mol·L^−1^ HSA_FR_ and HSA_INC_ absorption spectrum.

Figure 2 presents absorption spectra of human serum albumin (HSA). 

Based on Figure 2 it can be observed that HSA_INC_ isoform E absorption is higher than HSA_FR_. The absorption spectrum of protein in the range between 250 and 300 nm derives from phenylalanine, tyrosyl or/and tryptophanyl residues. In order to resolve the complex protein absorption spectrum into the individual contributions of the three aromatic amino acids, HSA_FR_ and HSA_INC_, second derivative of absorption spectra have been registered (Figure 2, insert). The second derivative of the spectrum shows how quickly the absorbance rate changes. The spectrum in the range between 250 and 270 nm illustrates the rate of change within phenylalanine residues. Changes in the range between 270 and 290 nm illustrate changes in tryptophanyl and tyrosyl residues environment, while changes in the spectrum in the range between 290 and 310 nm correspond to tryptophanyl residues. No differences in the course of absorption spectrum second derivative of HSA_FR_ and HSA_INC_ were recorded at 250 nm and about 300 nm wavelengths, which means that the concentration of both samples is the same. In the region between 253 and 298 nm significant differences were recorded, the smallest difference within the phenylalanyl residues, and the biggest difference within the tryptophanyl residue. Based on the data it can be concluded that the second derivative of HSA spectra has been employed for detecting conformational changes involving the microenvironments of aromatic amino acid residues of HSA_INC._ Juárez et al. recorded FT–IR spectra for HSA. According to the second derivative they observed the presence of a certain increase of disordered structure, also seen by far-UV CD. The appearance of a well-defined peak around 1625 cm^−1^ indicates a structural transformation to an intermolecular hydrogen-bonded-β-sheet structure, a structural characteristic of the amyloid fibrils [17].

### 3.3. Emission Fluorescence Spectra of Human Serum Albumin (HSA)

**Figure 3:**
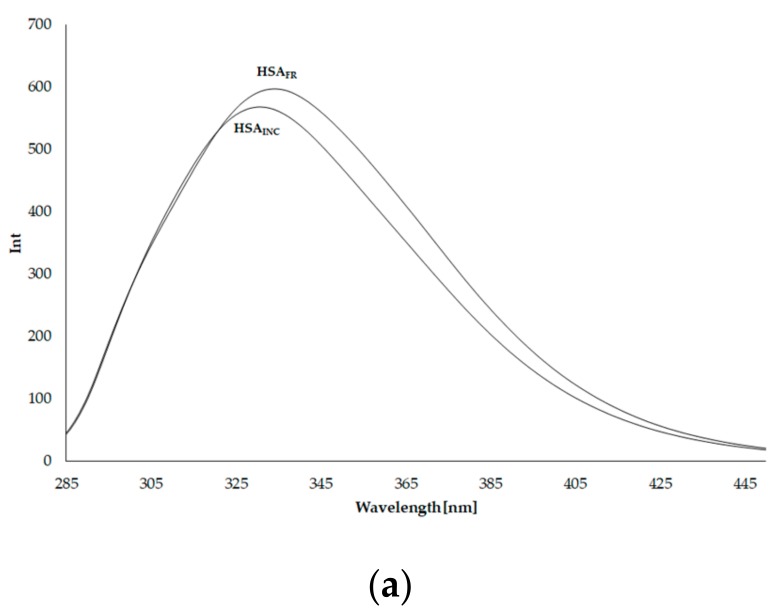

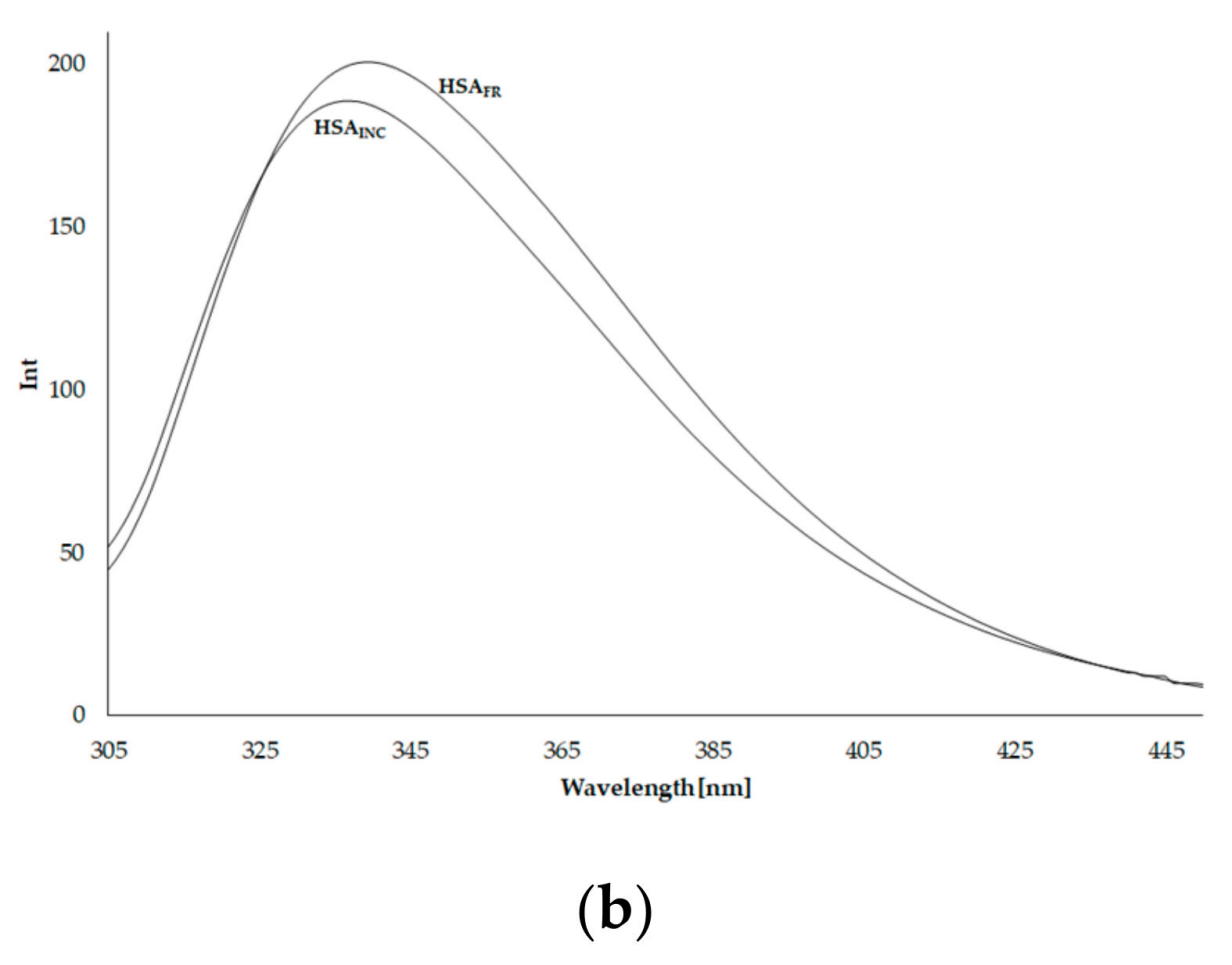
Emission fluorescence spectra of unmodified human serum albumin (HSA_FR_) and incubated human serum albumin (HSA_INC_) at 5 × 10^−6^ mol·L^−1^ concentration; (**a**) λ_ex_ 275 nm, (**b**) λ_ex_ 295 nm.

It is well known that the λ_ex_ 275 nm excitation wavelength results in fluorescence emission derived from tyrosyl and tryptophanyl residues, whereas the excitation wavelength of 295 nm causes fluorescence emission of tryptophanyl residue. Emission fluorescence spectra of HSA_FR_ and HSA_INC_, at excitation wavelengths λ_ex_ 275 nm and λ_ex_ 295 nm, respectively, are presented in Figure 3a,b.

A decrease in the fluorescence intensity has been attributed to tertiary structure changes, which cause the exposure of tryptophan to the solvent. The spectrum of aggregated HSA (HSA_INC_) is less intense than the spectrum of HSA_FR_ and shifts in the shortwave direction by 3 nm at excitation wavelength 275 nm and by 2 nm at 295 nm excitation. This indicates an increase in the hydrophobicity of the fluorophore environment: tyrosyl residues, especially tryptophanyl residue, which is very sensitive to the nature of the immediate environment. A reduction in tryptophanyl residue intensity accompanied by a slight blue shift (~3 nm reported also by Bhattacharya [16]) is a result of amyloid-like aggregates molding. Bhattacharya et al. suggested that the observed drop in fluorescence intensity could be either due to (i) exposure of tryptophanyl residue to the solvent and/or (ii) quenching of tryptophanyl residue intensity due to an increase in the number of the rest of phenylalanines, histidines, and disulfides in the proximity of tryptophanyl residue that quench the emission upon aggregation mediated by both electrostatic and hydrophobic interactions. The change in the conformation of HSA_INC_ relative to HSA_FR_ is confirmed by the reduction of full width at half maximum (FWHM) for HSA_INC_, both at excitation wavelength λ_ex_ 275 nm and λ_ex_ 295 nm. Data are collected in Table 2.

To confirm the short-term shift of the fluorescence band, the spectral parameter A has been calculated. Spectral parameter A ($A=\frac{F_{365\;\mathrm{nm}}}{F_{320\;\mathrm{nm}}}$) was used because of its sensitivity to small changes in the position of maximum fluorescence wavelength (λ_max_). The decrease in the value of parameter A for HSA_INC_ compared to HSA_FR_ confirms the shortwave spectrum shift. Shortwave fluorescence spectrum shift means that fluorophores (Tyr, Trp) of fibrillated HSA are in a more hydrophobic environment than HSA_FR_ fluorophores, which has been caused by the change in HSA conformation during heating for 9 days at 37 °C. Conformational changes of the protein after heating at 37 °C for 9 days, pH 2.0 (HSA_INC_), not only in subdomain IIA where tryptophanyl residue (Trp-214) is located, but also in other subdomains of protein containing tyrosyl residues (Tyr) were registered.

### 3.4. Synchronous Fluorescence Spectra of Human Serum Albumin (HSA)

**Figure 4:**
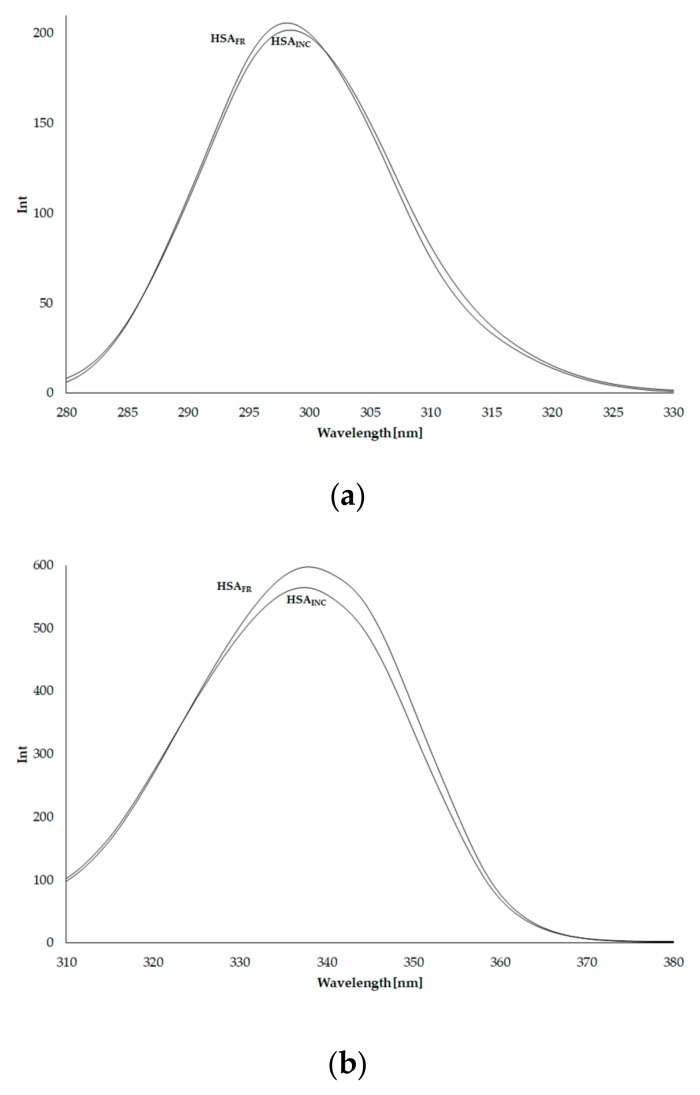
Synchronous fluorescence spectra of HSA_FR_ and HSA_INC_ at 5 × 10^−6^ mol·L^−1^ concentration; (**a**) ∆λ 15 nm, (**b**) ∆λ 60 nm.

To further study the changes in HSA_INC_ towards HSA_FR_, synchronous fluorescence spectra of human serum albumin (HSA) have been obtained and presented in Figure 4.

Synchronous fluorescence spectra of albumin allows observation of the fluorescence of tyrosyl (∆λ 15 nm) and tryptophanyl (∆λ 60 nm) residues, separately (Figure 4, Table 3).

At the same wavelength (λ_max_), maximum fluorescence of HSA_INC_ tyrosyl residues (∆λ 15 nm) is slightly less than the fluorescence of HSA_FR_ tyrosyl residues. This phenomenon confirms that tyrosyl residues are not very sensitive to changes in their environment. However, the decrease in fluorescence of HSA_INC_ tryptophanyl residue compared to HSA_FR_ is stronger and shortwave fluorescence band shift occurred, which agrees with the assumption that less-ordered, amyloid-like fibrils are formed in coexistence with amorphous aggregation [17]. This means that, in contrast to tyrosyl residues, the tryptophanyl residue is sensitive to changes in its surroundings, and the changes observed in the intrinsic tryptophan fluorescence intensity can be correlated to the protein conformational changes. The aggregation/fibrillation process is strictly dependent on external conditions.

### 3.5. Circular Dichroism (CD) Spectra of Human Serum Albumin (HSA)

**Figure 5:**
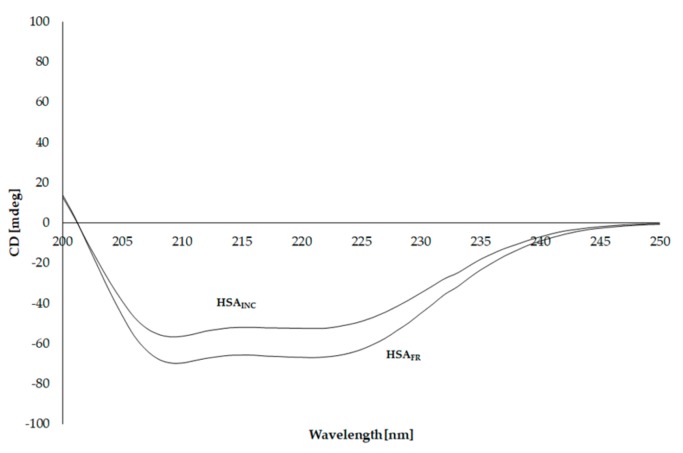
CD spectra of 3 × 10^−6^ mol·L^−1^ HSA_FR_ and HSA_IN__C_.

To investigate how changes at the secondary structure level are involved, CD measurements were performed. CD spectra of HSA_FR_ and HSA_INC_ are shown in Figure 5.

The observed HSA_FR_ ellipticity (mdeg]) illustrates that HSA is an α-helical protein. There are two clear minima in the CD spectra (Figure 5), at λ_min_ 210 nm and λ_min_ 220 nm (Table 4). Incubation of HSA at pH 2.0 for 9 days resulted in an increase in observed ellipticity (mdeg) and a shift of the band from 210 nm to 209 nm and 220 nm to 221 nm.

Based on the Equation (4) the mean residue ellipticity [Θ_MRW_], both for HSA_FR_ and HSA_INC_, has been calculated as shown in Table 4.

No changes observed in ellipticity (deg) at λ 200 nm and 250 nm have been registered. This phenomenon means that the concentration of both samples (HSA_FR_, HSA_INC_) is the same. For HSA_INC_ a Θ_MRW_ value is higher than for HSA_FR_, but at the second λ_min_ 221 nm this increase is stronger (1.27 times) than for the first one λ_min_ 209 nm (1.23 times). β-type proteins contain only one negative band around λ_min_ 220 nm. This stronger increase in the mean residue ellipticity for the 221 nm band indicates a decrease in the content of α-helical structures and an increase in β-structural elements. Due to the aggregation/fibrillation process HSA probably changes from the native state which was characterized by the presence of two minima at 221 and 209 nm with the existence of predominant α-helical structure to a spectrum with a visible minimum and almost invisible second one as a consequence of β-sheet structure (absent in native HSA [11,53]). The changes obtained in CD spectra suggest the increments of β-sheet and loop structures [46,54]. A significant fall in the mdeg values at 220 nm confirms substantial loss in the helical structure [18]. Bhattacharya et al. monitored the mechanism of aggregation and fibril formation from bovine serum albumin [16]. By the analysis of the far-UV CD spectra they also observed that after the sample incubation, there was a successive loss in the helicity, which may suggest that the formation of both β-sheet and unordered conformation occur at the expense of helical conformation. Juarez and Taboada also reported that the increase in random coil/β-sheet conformation is accompanied by a reduction in the α-helical content of protein structure [45]. A reduction in the α-helical content of protein structure and the formation of β-sheet conformation (the increase in **%**β-sheet value) have been confirmed using the Secondary Structure Estimation program with the Young’s reference model, presented in Table 5.

The aggregation process in protein can be governed by several factors such as temperature, environmental factors like general solution conditions, protein concentration, container/closure system and surfaces, light and irradiation [55], pH, presence of electrolytes, denaturants, and metal ions. Pandey et al. monitored the role of Cu(II) ions in inducing rapid fibrillation in human serum albumin via several spectroscopic methods like UV-Vis spectroscopy, fluorescence spectroscopy, CD, etc. [56]. They found that fibrillation is enhanced in the presence of Cu(II) ions and it plays a significant role in research relating to neurodegenerative diseases. Stirpe et al. studied the human serum albumin thermally-induced aggregation process in the presence of metal ions [57]. The presence of Cu(II) and Zn(II) ions was investigated by using optical absorption, fluorescence, AFM and EPR spectroscopy. They investigated the kinetics of HSA aggregation between 60 and 70 °C and based on the obtained results showed that the protein aggregates are elongated oligomers with fibrillar-like features. Stirpe et al. concluded that the presence of studied ions does not affect the thermally induced aggregation process or the morphology of HSA aggregates. 

### 3.6. HSA Binding Sites Assessment

It is well known and carefully examined that under in-vitro solution conditions where the native state is destabilized, many proteins present changed structure (abnormal structure) and their metabolism is associated with a strong tendency to self-aggregation into an amyloid polymeric fibril structure. This ability is a genetic feature of the polypeptide chains [11] and plays a key role in different pathogenesis of neurodegenerative diseases: Alzheimer’s, Parkinson’s or Creutzfeldt–Jakob. The formation of amyloid fibrils has been studied based on several techniques: fluorescence spectroscopy, UV-vis spectroscopy, CD spectroscopy, etc., and the possibility of changes in the secondary and tertiary structure has been reported. Human serum albumin plays a key role in the transport of exo- and endogenous substances and the changes in albumin binding sites can have a crucial role in the pharmacological effect. Thus, the study of amyloid fibril formation and their influence on the main binding sites is of great of importance.

#### 3.6.1. Dansyl-l-glutamine (dGlu) and Dansyl-l-proline (dPro)

In the HSA structure there are two main drug binding sites: IIA subdomain (Sudlow’s site I) and IIIA subdomain (Sudlow’s site II). It has been proven that IB subdomain is also drug binding site [58]. In order to assess the number and location of drug binding sites on the HSA molecule, dansylated amino acids have been used. The specificity of binding to a binding site of a given dansylated amino acid results from its structure. Amino acids containing hydrophobic side chains are characteristic for Sudlow’s site II (dansyl-l-Proline (dPro)), while those with side chains that are polar or those carrying an electric charge are characteristic for Sudlow’s site I (dansyl-l-Glutamine (dGlu)) [59].

**Figure 6:**
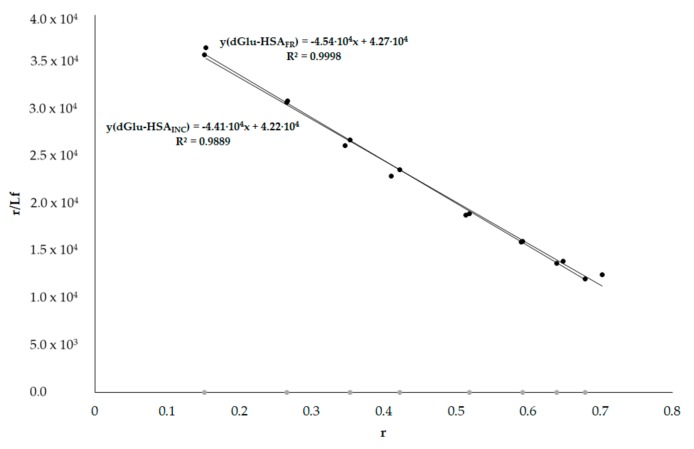
Scatchard curve for dGlu–HSA_FR_ and dGlu–HSA_INC_ systems; λ_ex_ 350 nm.

dGlu binds to HSA in Sudlow’s site I (IIA subdomain). By registering changes in dGlu fluorescence excited at 350 nm in the presence of HSA_FR_ and HSA_INC_, the association constant (K_a_) for dGlu in the presence of both HSA have been determined (Figure 6).

Using the data obtained for dGlu–HSA_FR_ and dGlu–HSA_INC_ complexes, the necessary parameters were determined to calculate the association constants K_a_ [mol^−1^·L]. For dGlu–HSA_FR_ association constants equal to K_a_ = 4.54 × 10^4^ mol^−1^·L and for dGlu–HSA_INC_ 4.41 × 10^4^ mol^−1^·L. Fluorescent label dGlu binds to IIA subdomain of HSA molecules. The lower value of dGlu association constant in the complex with HSA_INC_ compared to HSA_FR_ indicates that fibrillation changed the IIA subdomain of protein, however, the change in the K_a_ value is small.

**Figure 7:**
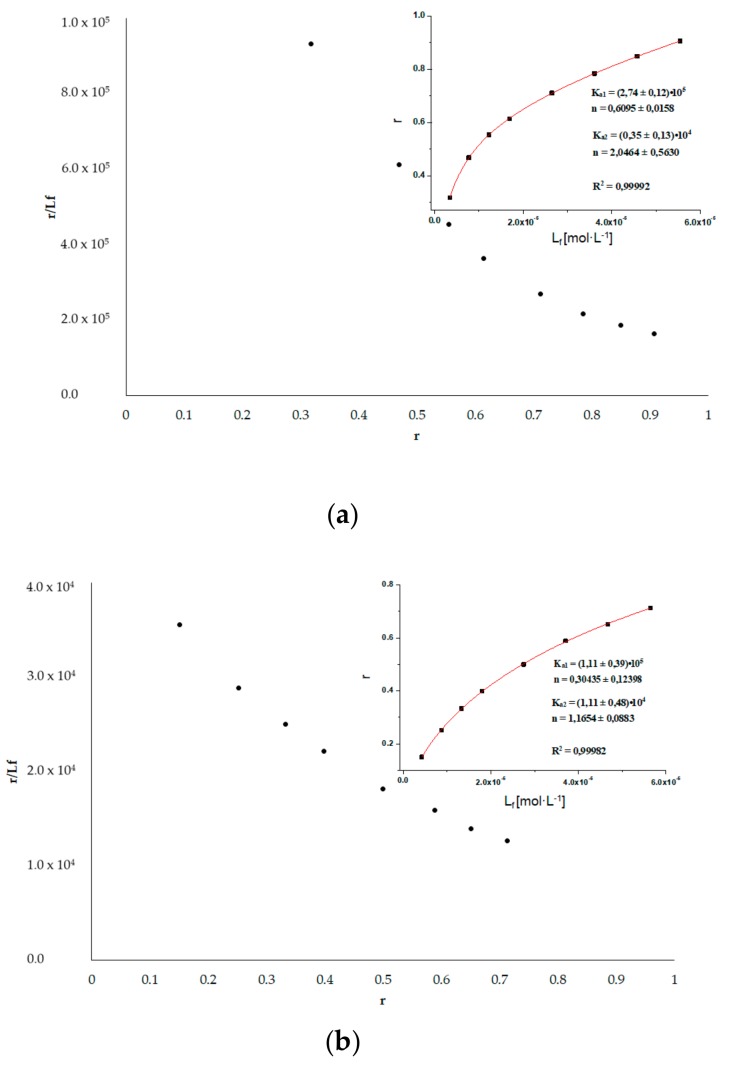
Scatchard curve for (**a**) dPro–HSA_FR_ and (**b**) dPro–HSA_INC_ systems. The inserts show the binding isotherms; λ_ex_ 350 nm.

dPro binds in the Sudlow’s site II (subdomain IIIA). From the registration of dPro emission fluorescence data (Figure not shown) excited at 350 nm, in the presence of HSA_FR_ and HSA_INC_, the Scatchard curves have been drawn (Figure 7) and the association constants K_a_ [mol^−1^·L] were calculated. The Scatchard plots obtained for dPro–HSA systems are not a straight line (Figure 7 insert), which means that there are more than one binding site for dPro in HSA subdomain. The association constants K_a_ [mol^−1^·L] were determined for two binding sites using the non-linear regression method based on the Levenberg–Marquardt algorithm (OriginPro 8.5 SR1 software).

By analyzing the association constants values calculated for dPro–HSA_FR_ (K_a1_ = (27.50 ± 1.20) × 10^4^ mol^−1^·L, K_a2_ = (0.35 ± 0.13) × 10^4^ mol^−1^·L) and dPro–HSA_INC_ (K_a1_ = (11.10 ± 3.90) × 10^4^ mol^−1^·L, K_a2_ = (1.10 ± 0.48) × 10^4^ mol^−1^·L) systems it can be concluded that the structure of HSA in IIIA subdomain has been changed by the incubation process for 9 days, pH 2.0, T = 37 °C, similar to the dGlu–HSA systems.

From dansyl amino acids–HSA measurements we confirmed that conformational changes in tertiary structure occur and both domain II and domain III are involved in the aggregation/fibrillation process.

#### 3.6.2. Phenylobutazone (Phb) and Ketoprofen (Ket)

**Figure 8:**
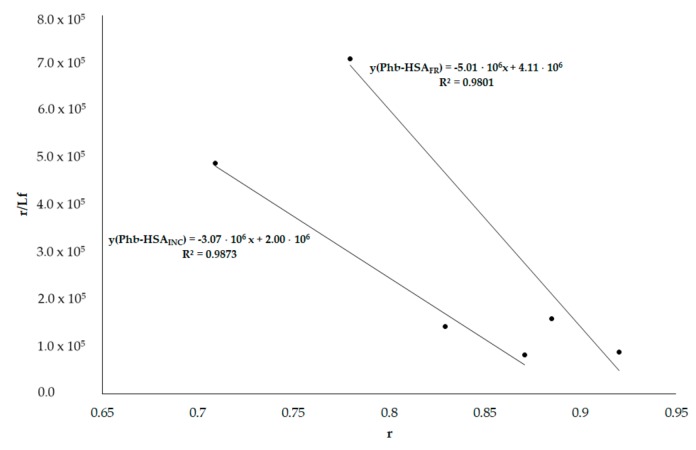
Scatchard curve for Phb–HSA_FR_ and Phb–HSA_INC_ systems; λ_ex_ 275 nm.

Phenylbutazone (Phb) binds to HSA subdomain IIA (Sudlow’s site I) while ketoprofen (Ket) binds to HSA subdomain IIIA (Sudlow’s site II). These drugs are recognized as binding sites markers. According to absorption spectra, Phb absorbs a light at 275 nm (used to excite HSA fluorescence) (data not shown). So as to not exceed the A_275 nm_ 0.3 value (the inner filter effects requirements), the Phb concentration was not higher than 1.5 × 10^−5^ mol·L^−1^. Using the data from emission fluorescence spectra of both HSA_FR_ and HSA_INC_ in the presence of Phb, at λ_ex_ 275 nm (data not shown), the Scatchard curves have been plotted (Figure 8).

Based on the Scatchard plots, the association constants K_a_ [mol^−1^·L] for Phb–HSA_FR_ and Phb–HSA_INC_ systems have been calculated and equal 5 × 10^6^ mol^−1^·L and 3 × 10^6^ mol^−1^·L, respectively. Lower values of K_a_ constants in Phb–HSA_INC_ relative to Phb–HSA_FR_ confirm the changes in subdomain IIA of fibrillated HSA.

**Figure 9:**
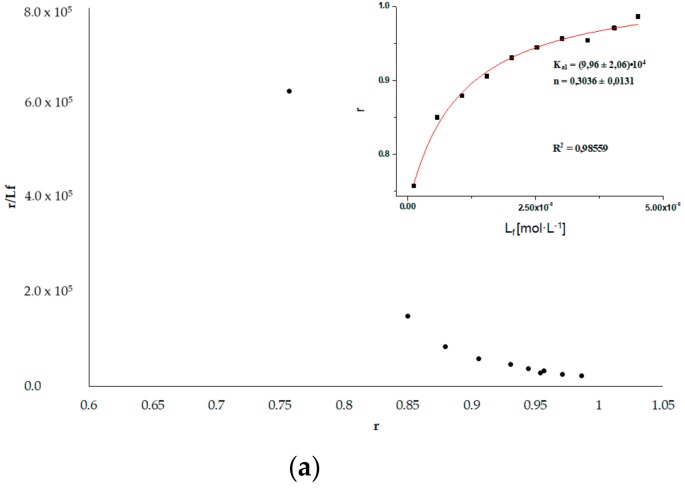

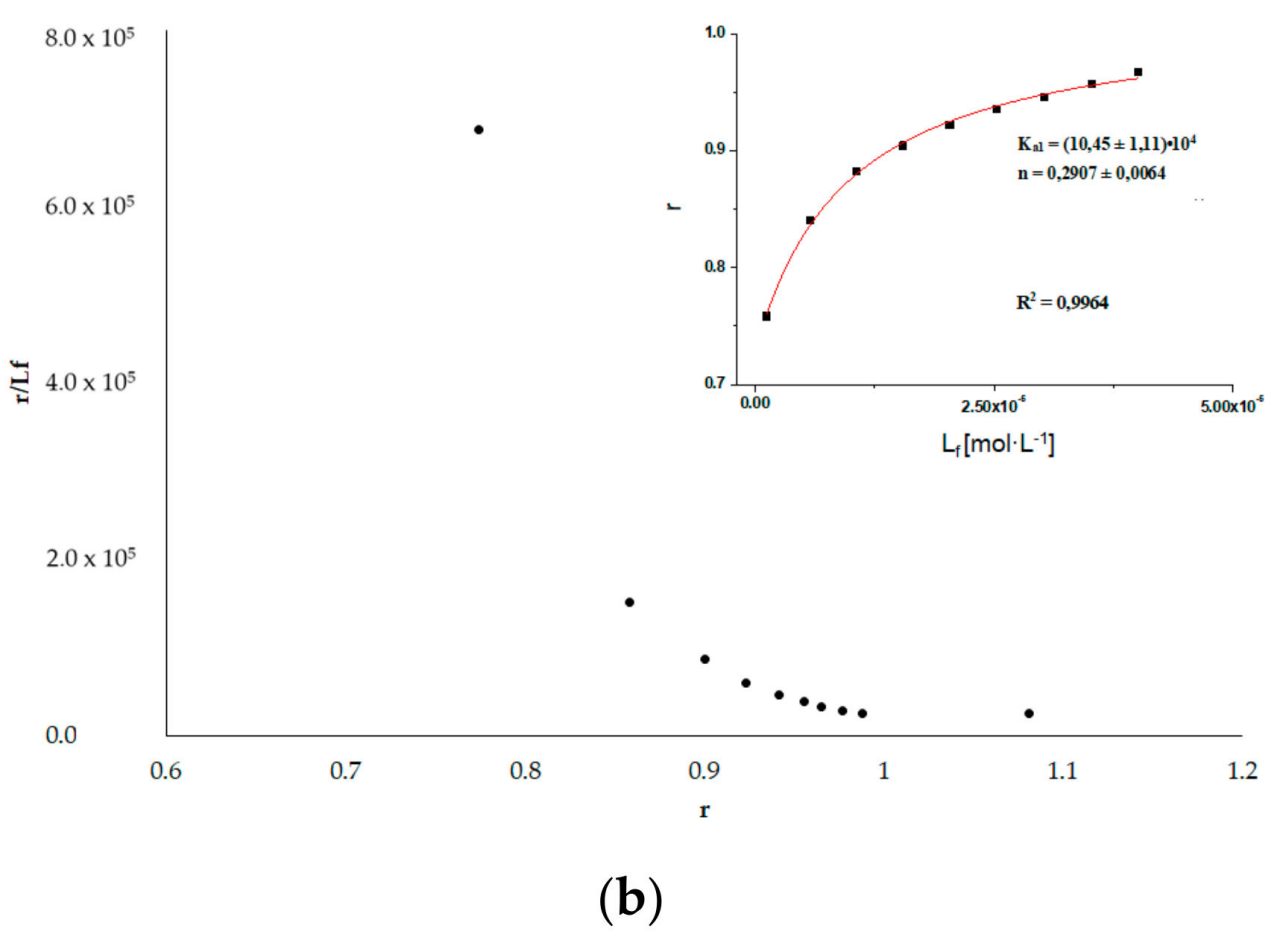
Scatchard curve for (**a**) Phb–HSA_FR_ and (**b**) Phb–HSA_INC_ systems. Inserts show the binding isotherms; λ_ex_ 295 nm.

Figure 9 presents the Scatchard plots and binding isotherms for the excitation wavelength λ_ex_ 295 nm (emission fluorescence spectra at λ_ex_ 295 nm, not shown).

The Scatchard plots obtained for Phb–HSA_FR_ and Phb–HSA_INC_ systems at λ_ex_ 295 nm are not linear. This phenomenon means that there are more than one binding site for Phb in subdomain IIA of both HSA. It is known that subdomain IIA was divided by Yamasaki [7,60] into three regions: Ia, Ib and Ic. Phb probably binds to region Ib and partly to region Ia. For two classes of binding sites the association constants K_a_ [mol^−1^·L] were determined based on the binding isotherms using the non-linear regression method (Levenberg–Marquardt algorithm using the computer program OriginPro 8.5 SR1 – Northampton, MA, USA).

By analyzing the association constants values calculated for Phb–HSA_FR_ (K_a1_ = (9.96 ± 2.06) × 10^4^ mol^−1^·L, K_a2_ unachievable) and Phb–HSA_INC_ (K_a1_ = (10.45 ± 1.11) × 10^4^ mol^−1^·L, K_a2_ unachievable) systems at λ_ex_ 295 nm it can be confirmed that the structure of HSA in subdomain IIA has been changed by the incubation process (aggregation/fibrillation) for 9 days, pH 2.0, T = 37 °C. It is noteworthy that the association constants obtained in dGlu–HSA_FR_ and dGlu–HSA_INC_ complexes differ from the values obtained in Phb–HSA_FR_ and Phb–HSA_INC_ complexes. 

**Figure 10:**
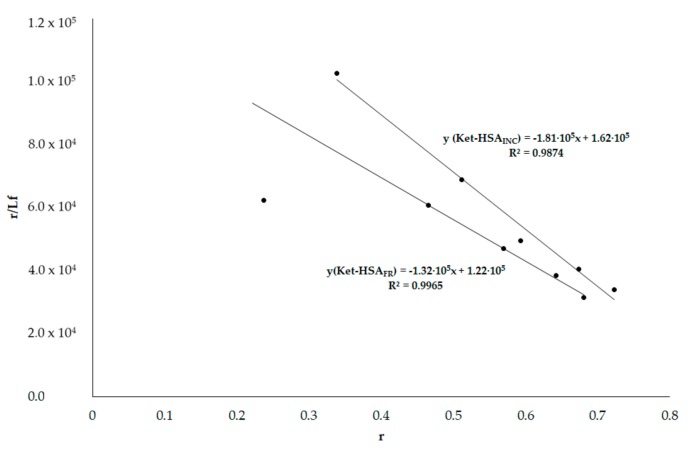
Scatchard curve for Ket–HSA_FR_ and Ket–HSA_INC_ systems; λ_ex_ 275 nm.

**Figure 11:**
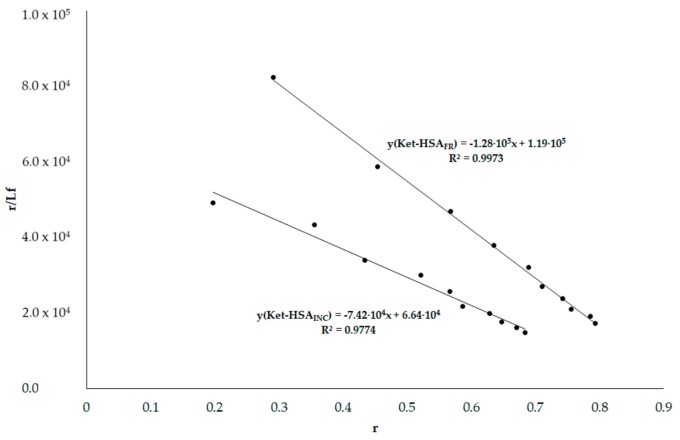
Scatchard curve for Ket–HSA_FR_ and Ket–HSA_INC_ systems; λ_ex_ 295 nm.

Similarly, as for the systems of Phb–HSA, based on Ket absorption spectra it can be concluded that Ket absorbs light at 275 nm (used to excite HSA fluorescence) (data not shown). Similarly as in the case of Phb, so as to not exceed the A_275 nm_ 0.3 value (inner filter effects requirements), the Ket concentration was no higher than 2.5 × 10^−5^ mol·L^−1^. Using the data from emission fluorescence spectra of HSA_FR_ and HSA_INC_ at λ_ex_ 275 nm and 295 nm (data not shown) in the presence of Ket, the Scatchard curves have been plotted (Figure 10 and Figure 11) and the association constants have been calculated.

Based on the Scatchard curves, the association constants values calculated for Ket–HSA_FR_ and Ket–HSA_INC_ systems are 13.2 × 10^4^ mol^−1^·L and 18.1 × 10^4^ mol^−1^·L, respectively. Figure 11 presents Scatchard curves in the same systems as above, but at the excitation wavelength λ_ex_ 295 nm.

Calculated association constant values for Ket–HSA_FR_ and Ket–HSA_INC_ systems at λ_ex_ 295 nm are 12.80 × 10^4^ mol^−1^·L and 7.42 × 10^4^ mol^−1^·L, respectively, and the influence of aggregation/fibrillation on HSA structure has been elucidated.

## 4. Conclusions

Knowledge derived from the physicochemical analysis of protein fibrillation/aggregation helps to understand an important and growing category of diseases in terms of life quality and expectancy. The conducted research carried out for HSA_FR_ and HSA_INC_ and their systems with ligands allowed us to obtain very promising conclusions. HSA incubated for 7/9 days at pH 2.0 and T = 37 °C causes conformational changes in the structure of the molecule, and previously absent β-structural aggregates/fibrillates are formed. This is important because HSA does not contain β-structures in its overall structure. Changes in the conformation of the molecule that occur during incubation affect its ability to bind ligands. Surroundings of cavities in which Sudlow’s sites I and II are located become more hydrophobic. The conformational changes inducing partial exposure of hydrophobic residues have a common feature in being the step for initiating intermolecular cross-linking interactions. The decrease in the association constant values K_a_ [mol^−1^·L] calculated for HSA_INC_ systems with dansyl glutamine, dansyl proline, phenylbutazone and ketoprofen relative to the complexes with HSA_FR_ confirms the conformational changes of aggregated/fibrillated albumin. Moreover its aggregation/fibrillation adversely affects drug-transport ability and monitoring therapy should be taken into account. Therefore, understanding the mechanism of fibrillation/aggregation of HSA and potentially the design of suitable inhibitor molecules for stabilizing its native conformation, are of utmost importance. Furthermore, the obtained results taken together with other data can elucidate the mechanism of fibril formation and changes in protein binding sites that would be useful in the design of antiamyloid therapeutics. 

## Figures and Tables

**Table 1:** ThT fluorescence (1 × 10^−5^ mol·L^−1^) in the absence and presence of HSA_FR_ and HSA_INC_ at 1 × 10^−6^ mol·L^−1^ concentration, λ_ex_ 440 nm.

System	λ_max_ [nm]	F_cor_
ThT	477	29.032
ThT–HSA_FR_	476	47.185
ThT–HSA_INC_ (incubated at 37 °C for 9 days)	477	94.624
ThT–HSA_INC_ (incubated at 65 °C for 7 days)	476	797.720

**Table 2:** Fluorescence of unmodified human serum albumin (HSA_FR_) and incubated human serum albumin (HSA_INC_) at 5 × 10^−6^ mol·L^−1^ concentration; the excitation wavelength λ_ex_ 275 nm and λ_ex_ 295 nm.

5 × 10^−6^ mol·L^−1^	λ_ex_ 275 nm				λ_ex_ 295 nm			
	λ_max_ (nm)	F	Parameter A	FWHM (nm)	λ_max_ (nm)	F	Parameter A	FWHM (nm)
HSA_FR_	334	596.771	0.785	73.351	339	200.726	1.126	60.698
HSA_INC_	331	567.627	0.673	70.217	337	188.772	0.954	57.787

**Table 3:** Intensity of 5 × 10^−6^ mol·L^−1^ HSA_FR_ and HSA_INC_ synchronous fluorescence spectra.

5 × 10^−6^ mol·L^−1^	∆15 nm		∆60 nm	
	λ_max_ (nm)	F_cor_	λ_max_ (nm)	F_cor_
HSA_FR_	298	205.72	338	597.00
HSA_INC_	298	201.66	337	564.46

**Table 4:** [Θ_MRW_] at λ_min_ for 3 × 10^−6^ mol·L^−1^ HSA_FR_ and HSA_INC_.

3 × 10^−6^ mol·L^−1^	λ_min_ [nm]	[Θ_MRW_] $\left[deg\cdot cm^{2}\cdot dmol^{-1}\right]$	λ_min_ [nm]	[Θ_MRW_] $\left[deg\cdot cm^{2}\cdot dmol^{-1}\right]$
**HSA_FR_**	210	−19825.20	220	−19012.80
**HSA_INC_**	209	−16118.50	221	−14941.03

**Table 5:** The percentage (%) content of the secondary structure elements of HSA_FR_ and HSA_INC_.

Albumin	% α-Helix	% β-Sheet	% Other
**HSA_FR_**	35.2	8.4	56.4
**HSA_INC_**	32.9	11.9	55.2
